# Early feeding practices and body mass index z-score among Saudi preschoolers: a cross-sectional study

**DOI:** 10.1186/s12887-022-03666-8

**Published:** 2022-10-20

**Authors:** Rana H. Mosli

**Affiliations:** Clinical Nutrition Department, Faculty of Applied Medical Sciences, King Abdulaziz University, Jeddah, Saudi Arabia

**Keywords:** Early feeding Practices, Complimentary feeding, Birthweight, Body Mass Index Z-Score, Birthweight

## Abstract

**Background:**

Feeding practices during early years may have long-lasting influences on eating behaviors, growth patterns and body mass index (BMI) trajectory. The objectives of this study were to characterize early feeding practices among mothers in Saudi Arabia and examine their associations with child birthweight and BMI z-score (BMIz) at preschool.

**Methods:**

This is a cross-sectional study including 209 mother-child dyads who were recruited from different preschools around the city of Jeddah, Saudi Arabia. Mothers completed the study questionnaire over the telephone and preschoolers’ anthropometric measurements were objectively measured using standardized procedures. Primary predictors included variables pertaining to breastfeeding initiation, breastfeeding duration, formula milk introduction, complementary feeding, and offering fruit juice, date syrup-milk mixture, and soda drinks in a baby bottle. The study’s primary outcome was BMIz at preschool. Mothers reported child’s birthweight and sociodemographic characteristics. Descriptive statistics were used to characterize early feeding practices. Bivariate analyses and linear regression analysis were used to examine the association of early feeding practices with child birthweight and BMIz at preschool.

**Results:**

About half of the mothers reported that they have offered fruit juice and/or date syrup-milk mixture in a baby bottle (52.2% and 45.9% respectively), with an average duration of 11.5 months (SD = 7.73) and 5.90 months (SD = 6.13), respectively. Children who were offered fruit juice and/or date syrup-milk mixture in a baby bottle had significantly lower birthweights compared to children who were not (M = 2.79, SD = 0.59 vs. M = 3.06, SD = 0.69, *P* < 0.01 and M = 2.79, SD = 0.67 vs. M = 3.03, SD = 0.62, *P* < 0.01, respectively). There was a negative association between introducing fruit juice in a baby bottle and child BMIz at preschool (β: -0.18, 95% confidence interval (CI): -0.83, -0.11); This association was not significant after adjusting for child birthweight and other covariates (β: -0.10, 95% CI: -0.64, 0.09).

**Conclusion:**

A large proportion of mothers reported offering fruit juice and date syrup-milk mixture in a baby bottle. Additional research is needed to understand associations with child birthweight and BMIz. Longitudinal and interventions studies can help inform counseling guidelines and community campaigns in order to improve early feeding practices in the region.

## Introduction

Feeding practices during early years may have long-lasting influences on eating behaviors, growth patterns and body mass index (BMI) trajectory [1, 2]. Early feeding practices in the Middle East have been widely explored [3]. Mothers in the region have shown strong commitment to breastfeeding as a result of religious beliefs; On average, two-thirds of children are breastfed beyond the first year of life [4, 5] However, early complementary feeding is common among Middle Eastern/Arab mothers stemming from cultural and familial traditions [4]. On average, only around 24% of infants in the region are exclusively breastfed [5, 6]. First complementary foods include juice and date products (e.g., date syrup mixed with milk), which are often introduced within the first few weeks of life [4, 7].

Current recommendations regarding fruit juice consumption among infants state that fruit juice offer no additional nutritional benefits and should not be introduced prior to one year of age unless clinically indicated [8]. For toddlers between one and three years of age, fruit juice should not be offered in bottles or easily transportable cups, and intake should not exceed four ounces per day [8]. The growing concern surrounding juice intake in children is related to it being easily overconsumed when offered, and to the negative consequences of overconsumption [8, 9]. Overconsumption of fruit juice can lead to displacement and insufficient intake of nutrients from other food sources, including protein and calcium from milk, as well as short stature, excessive sugar intake, diarrhea, tooth decay and pneumonia [10–14]. Evidence surrounding the association between fruit juice consumption and overweight risk among children is conflicting [15]. While one study reported that children who consistently drink juice at two years of age might have higher odds of overweight at age four [16], others found that consumption of fruit juice is associated with minimal, non-clinically significant weight gain in children ages one to six years [17].

Offering sugary liquids such as fruit juice in baby bottles has been observed among a large proportion (73%) of a cohort of Hispanic mothers [18]. However, this practice has not been adequately evaluated in Middle Eastern/Arab populations. Feeding infants from a bottle might be associated with greater liquid intake compared to when infants are fed from the breast or a cup [19]. Thus, offering sugary liquids, such as juice and date syrup-milk mixture, in bottles might lead to overconsumption. Furthermore, mothers have previously reported that their child’s birthweight may affect their decisions regarding solid food introduction [20]. However, the association between child’s birthweight and offering sugary liquids in baby bottles has not been sufficiently explored.

The current literature is lacking data regarding the prevalence of offering sugary drinks (e.g., fruit juice and date syrup-milk mixture) in bottles in Middle Eastern/Arab countries such as Saudi Arabia (SA). Furthermore, the association of intake of sugary drinks during infancy and offering these drinks in baby bottles with child birthweight and body mass index (BMI) at preschool is underdeveloped. The objective of this study was to characterize early feeding practices among a sample of mothers in SA and examine their association with child birthweight and child BMI at preschool while adjusting for various covariates.

## Methods

### Study protocol

A total of 209 mother-child dyads were recruited through cluster sampling from different preschools around the city of Jeddah, SA that were randomly selected based on location; Two preschools were located in the Eastern area, two in the Western area, two in the Northern area, and two in the Southern area. Four of the eight preschools were government-subsidized centers, and four were private preschools. An invitation to participate in the study was sent to all mothers via their children’s backpacks. The invitation letter included a description of the study, screening items that assessed eligibility for participation, and the participation consent form. Mothers who were eligible to participate and returned signed consent forms were contacted by research assistants who completed the study questionnaire with them over the telephone. The study questionnaire was administered in Arabic and included questions that assessed early feeding practices and sociodemographic characteristics. The study questionnaire was pretested using a different sample consisting of 60 mothers of preschool children and was reviewed by an expert committee of four health care professionals. Minor clarifications were made to wording of questions based on comments from mothers and expert committee members.

About a week following questionnaire completion, the study team visited the preschools in order to obtain anthropometric measurements from children enrolled in the study. Study inclusion criteria were: Child is three to five years of age and is healthy with no serious medical problems or history of food allergies; Mother is fluent in Arabic. Ethical approval to conduct this study was obtained from the Unit of Biomedical Ethics at King Abdulaziz University (reference number HA-02-J-008).

### Measures

#### Early feeding practices

Mothers were asked if they had ever breastfed their child, followed by questions regarding the duration of breastfeeding, and the timing at which formula milk was introduced, if ever. Mothers were then asked questions regarding complementary feeding and the timing at which any foods or drinks other than milk and water were introduced. Then, mothers were asked the following regarding fruit juice, date syrup-milk mixture, and soda drinks: (1) If the drink was ever introduced in a baby bottle, (2) the child’s age at which the drink was first offered in a baby bottle, and (3) the child’s age at which offering the drink in a baby bottle was discontinued. The child’s age at which the drink was first offered in a baby bottle and age at which offering the drink in a baby bottle was discontinued were later used to calculate “duration of offering drink in bottle”.

#### Child BMI z-score (BMIz)

Children’s weights and heights were measured by trained research assistants using standardized procedures. BMI was calculated for each child as weight (kg) divided by height2 (m2) [21]. The age- and sex-specific World Health Organization (WHO) growth standards and reference data were used to calculate BMIz for each child [22, 23]. Weight and height data were missing for two children who have transferred to other schools, and these participants were therefore excluded from analyses involving child BMIz.

#### Birthweight and sociodemographic characteristics

Mothers reported their child’s birthweight as well as data regarding sociodemographic characteristics, which included information about the child’s sex and birthdate, the mother’s birthdate, educational level, and employment status, and the family’s total monthly income.

( ≤ 10,000 SR vs. > 10,000 SR) (10,000 SR is equivalent to 2,666 USD) [24]. Child and maternal age were calculated based on birthdates and dates of interviews.

### Statistical analysis

Analyses were conducted using IBM SPSS Statistics 21.0 (Armonk, NY, USA). Descriptive statistics, including means and standard deviations and frequencies and percentages, were used to describe the sample and early feeding practices. Pearson correlations were used to examine the association between various early feeding practices (e.g., breastfeeding duration and age of formula milk introduction).

Bivariate analyses, including independent samples t-test and Pearson correlations were used to examine the association between early feeding practices and child birthweight, and between early feeding practices and child BMIz at preschool. Then, in order to examine the adjusted association between early feeding practices and child BMIz at preschool, linear regression models were conducted. Only feeding practices that were significantly associated with child BMIz in bivariate analyses were inserted as primary predictors in regression models. Separate regression models were conducted for each primary predictor, and adjusted R^2^ was calculated for each model. Regression models were adjusted for child birthweight, child sex, breastfeeding duration maternal education, and total monthly income, each of which were previously found to be associated with both early complementary feeding and child BMI [20, 25–28].

## Results

### Sample characteristics

Description of sample characteristics is shown in Table 1. Mean child birthweight was 2.93 (SD = 0.65) and mean child BMIz at preschool was 1.17 (SD = 1.34). About half of the sample (51.7%) was male, and mean child age was 4.79 years (SD = 0.79). More than half of participating mothers (68.4%) had a college degree or higher, and around half (51.2%) were housewives. About half of the sample (45.5%) were considered “low-income”, as they reported to have a total monthly income of 10,000 SR or less.

**Table 1:** Sample Characteristics (n = 209)^*^

Variable	
Child Birthweight, M (SD)	2.93 (0.65)
Child BMIz at Preschool, M (SD)	1.17 (1.34)
Child Sex, N (%) Male Female	108 (51.7) 101 (48.3)
Child Age, M (SD)	4.79 (0.79)
Maternal Age, M (SD)	33.05 (4.98)
Maternal Education, N (%) < College degree ≥ College degree	66 (31.6) 143 (68.4)
Maternal Employment, N (%) Employed Housewife Student Other	84 (40.2) 107 (51.2) 15 (7.20) 3 (1.40)
Total Monthly Income, N (%) ≤ 10,000 SR > 10,000 SR	95 (45.5) 114 (54.5)

^*^ Table showing means (M) and standard deviations (SD) or counts (n) and percentages (%)

### Description of early feeding practices

The majority of mothers in our sample (92.3%) initiated breastfeeding, with an average breastfeeding duration of 9.34 months (SD = 8.04). The average age at which formula milk and solid foods were introduced was 2.89 months (SD = 5.44) and 5.93 months (SD = 2.11), respectively. Mean child age at which fruit juice was introduced was 8.17 months (SD = 5.93), with 52.2% (n = 109) of mothers reporting that they have offered fruit juice in a baby bottle at least once. Mean child age at which fruit juice was first offered in a baby bottle was 6.45 months (SD = 2.28), and the average duration for offering fruit juice in a baby bottle was 11.5 months (SD = 7.73). Only 1.00% (n = 2.00) of mothers reported offering a soda drink in a baby bottle at least once, while 45.9% (n = 96.0) reported offering date syrup-milk mixture in a baby bottle at least once. The average duration for offering date syrup-milk mixture in a baby bottle was 5.90 months (SD = 6.13) (Table 2).

**Table 2:** Description of Early Feeding Practices*

Variable	
Ever breastfed, n (%) Yes No	193 (92.3) 16.0 (7.70)
Breastfeeding Duration, M (SD) (n = 199)	9.34 (8.04)
Age of formula introduction, M (SD) (n = 173)	2.89 (5.44)
Age of solid food introduction, M (SD)	5.75 (2.11)
Age of fruit juice introduction, M (SD) (n = 199)	8.17 (5.93)
Ever offered fruit juice in bottle, n (%) Yes No	109 (52.2) 100 (47.8)
Age at which fruit juice was introduced in bottle (n = 109)	6.45 (2.82)
Duration of offering fruit juice in bottle (n = 109)	11.5 (7.73)
Ever offered soda drink in bottle, n (%) Yes No	2.00 (1.00) 207 (99.0)
Ever offered date syrup-milk mixture in bottle, n (%) Yes No	96.0 (45.9) 113 (54.1)
Age at which date syrup-milk mixture was introduced in bottle, M (SD) (n = 96)	3.22 (3.02)
Duration of offering date syrup-milk mixture in bottle, M (SD) (n = 96)	5.90 (6.13)

^*^Sample size equal to 209 unless otherwise indicated. Age and duration measured in months

Breastfeeding duration was positively correlated with age of formula milk introduction; introducing formula milk later was associated with longer breastfeeding duration (r = 0.53, *P* < 0.01). Age of formula milk introduction was positively correlated with duration of offering fruit juice in a baby bottle (r = 0.21, *P* < 0.05). Age of solid food introduction was positively correlated with age of fruit juice introduction (r = 0.29, *P* < 0.01), age at which fruit juice was introduced in a baby bottle (r = 0.45, *P* < 0.01), as well as age at which date syrup-milk mixture was introduced in a baby bottle (r = 0.26, *P* < 0.05). Age of fruit juice introduction was positively correlated with age at which fruit juice was introduced in a baby bottle (r = 0.84, *P* < 0.01). Furthermore, age at which date syrup-milk mixture was introduced in a baby bottle was positively correlated with duration of offering date syrup-milk mixture in a baby bottle (r = 0.25, *P* < 0.05) (Table 3).

**Table 3:** Association between Various Early Feeding Practices Assessed by Pearson Correlation

	Breastfeeding Duration	Age of formula introduction	Age of solid food introduction	Age of fruit juice introduction	Age at which fruit juice was introduced in bottle	Duration of offering fruit juice in bottle	Age at which date syrup-milk mixture was introduced in bottle	Duration of offering date syrup-milk mixture in bottle
Breastfeeding Duration	-							
Age of formula introduction	0.53^**^	-						
Age of solid food introduction	0.06	0.06	-					
Age of fruit juice introduction	-0.01	-0.14	0.29^**^	-				
Age at which fruit juice was introduced in bottle	-0.02	-0.01	0.45^**^	0.84^**^	-			
Duration of offering fruit juice in bottle	0.15	0.21^*^	-0.15	-0.04	-0.06	-		
Age at which date syrup-milk mixture was introduced in bottle	-0.15	-0.06	0.26^*^	0.07	-0.08	-0.08	-	
Duration of offering date syrup-milk mixture in bottle	0.08	0.16	0.03	0.02	0.23^**τ**^	0.01	0.25^*^	-

^τ^ P-value < 0.10 ^*^P-value < 0.05 ^**^P-value < 0.01

### Associations of early feeding practices with child birthweight and BMIz at preschool

- Bivariate Analysis.

Bivariate analysis showed that children who were offered fruit juice in a baby bottle had a significantly lower birthweight compared to children who were never offered fruit juice in a baby bottle (M = 2.79, SD = 0.59 vs. M = 3.06, SD = 0.69, *P* < 0.01). Likewise, children who were offered date syrup-milk mixture in a baby bottle had a significantly lower birthweight compared to children who were never offered date syrup-milk mixture in a baby bottle (M = 2.79, SD = 0.67, and M = 3.03, SD = 0.62, *P* < 0.01). Children who were offered fruit juice in a baby bottle had a significantly lower BMI z-score at preschool compared to children who were never offered fruit juice in a baby bottle (M= -0.06, SD = 1.41 vs. M = 0.42, SD = 1.22, *P* < 0.05). There was no significant association between offering date syrup-milk mixture in a baby bottle and BMI z-score at preschool (Table 4).

**Table 4:** Bivariate Association of Early Feeding Practices with Child Birthweight and BMIz at Preschool^1^

	Association with Child Birthweight	Association with BMIz at Preschool
*Differences in Means*		
**Ever breastfed** **Yes** **No**	2.95 (0.65) 2.63 (0.67)	0.18 (1.31) -0.35 (1.47)
**Ever offered fruit juice in bottle** **Yes** **No**	2.79 (0.59)^**^ 3.06 (0.69)	-0.06 (1.41)^*^ 0.42 (1.22)
**Ever offered date syrup-milk mixture in bottle** **Yes** **No**	2.79 (0.62)^**^ 3.03 (3.03)	0.05 (1.22) 0.27 (1.43)
*Pearson Correlation*		
**Breastfeeding Duration**	0.14^*^	0.09
**Age of formula introduction**	0.00	-0.01
**Age of solid food introduction**	0.07	0.19^**^
**Age of fruit juice introduction**	0.26^**^	0.18^*^
**Age at which fruit juice was introduced in bottle**	-0.22	-0.02
**Duration of offering fruit juice in bottle**	-0.05	-0.13
**Age at which date syrup-milk mixture was introduced in bottle**	-0.09	0.11
**Duration of offering date syrup-milk mixture in bottle**	-0.11	-0.10

^1^Body mass index z-score (BMIz). Differences in means examined using independent samples t-test

Breastfeeding duration and age of fruit juice introduction were each positively correlated with child birthweight (r = 0.14, *P* < 0.05, and r = 0.16, *P* < 0.01, respectively). Age of solid food introduction and age of fruit juice introduction were each positively correlated with BMI z-score at preschool (r = 0.19, *P* < 0.01, and r = 0.18, *P* < 0.05, respectively).

b. Regression Analysis

Regression analysis showed that offering fruit juice in a bottle was associated with lower child BMIz at preschool (β: -0.18, 95% confidence interval (CI): -0.83, -0.11). However, this association was not significant after adjusting for child birthweight, child sex, breastfeeding duration, maternal education, and family monthly income (β: -0.10, 95% CI: -0.64, 0.09). Age of solid food introduction was associated with higher BMIz at preschool (β: 0.19, 95% CI: 0.03, 0.20), and this associations did not meaningfully change after adjusting for covariates (β: 0.18, 95% CI: 0.03, 0.18). Furthermore, age of fruit juice introduction was associated with higher BMIz at preschool (β: 0.18, 95% CI: 0.00, 0.06). However, this association was not significant after adjusting for covariates (β: 0.12, 95% CI: -0.00, 0.06) (Table 5).

**Table 5:** Unadjusted and Adjusted Associations between Early Feeding Practices and BMIz at Preschool^1^

	Unadjusted Association with BMIz at Preschool Standardized beta (95% CI)	Adjusted Association with BMIz at Preschool^2^ Standardized beta (95% CI)	Adjusted R^2^
**Ever offered fruit juice in bottle** **Yes** **No**	-0.18 (-0.83, -0.11)^**^ 1	-0.10 (-0.64, 0.09) 1	0.10
**Age of solid food introduction**	0.19 (0.03, 0.20)^**^	0.18 (0.03, 0.19)^**^	0.12
**Age of fruit juice introduction**	0.18 (0.00, 0.06)^**^	0.12 (-0.00, 0.06)	0.10

^1^Body mass index z-score (BMIz)

^2^Regression models adjusted for child birthweight, child sex, breastfeeding duration, maternal education, and total monthly income

^**^P-value < 0.01

## Discussion

This observational study explored early feeding practices among a sample of 209 Saudi mothers and examined associations with child birthweight and BMIz at preschool. As previously reported in other countries in the region, prevalence of breastfeeding initiation in our sample (92.3%) is relatively high, and average breastfeeding duration (9.34 months, SD = 8.04) is longer than what was reported elsewhere [5, 6, 29–31]. On the other hand, the prevalence of offering sugary liquids in a baby bottle is concerning; About half of mothers in our sample reported offering fruit juice and date syrup-milk mixture in a baby bottle at least once. However, a higher percentage of mothers (73%) reported adhering to this practice in El Salvador [18]. The average duration of consuming fruit juice and date syrup-milk mixture in a baby bottle is also troubling (11.5 and 5.90 months on average, respectively), since offering sugary drinks in a baby bottle has been previously associated with overconsumption, excessive sugar intake, diarrhea and tooth decay [8, 10–13].

In line with findings from previous studies, our study found that introducing formula milk at an older age was associated with longer breastfeeding duration [32]. We also found that introducing formula milk at an older age was associated with consumption of fruit juice in a bottle for a longer duration and introducing date syrup-milk mixture in a baby bottle at an older age was associated with consuming it in a bottle for a longer duration. This suggests that older infants may develop a stronger preference for consuming various types of sugary liquids in a baby bottle compared to younger infants. Previous research has shown that flavor programming among infants and preference for liquids is linked to infants’ age [33]. Additional research is needed to better understand the intercorrelation of various feeding practices and their interaction with child’s age.

In our sample, children who were offered fruit juice or date syrup-milk mixture in a baby bottle had a lower birthweight. Birthweight has been also linked to early feeding practices in previous studies. However, cultural variations may exist; For example, higher birthweight was found to predict later initiation of complementary feeding in the US [28]. While in European populations, pretem birth was associated with earlier complementary feeding [34]. It is unknown whether mothers in the Middle Eastern region opt to introduce sugary liquids in bottles to lower birthweight infants in order to provide additional calories. Further qualitative studies are needed to assess caregivers’ beliefs and perceptions towards various early feeding practices in order to inform more effective, culturally-sensitive intervention programs.

Unadjusted analyses showed that offering fruit juice in a baby bottle was associated with lower BMIz at preschool. However, adjusting this association for child birthweight and other covariates resulted in the association becoming non-significant, indicating that it was at least partly explain by the covariates. Additionally, our results showed that age of fruit juice introduction was positively associated with child BMIz at preschool. However, this association also became non-significant after adjusting for covariates. Therefore, the association between later fruit juice introduction and higher BMIz at preschool could be explained by the child having a higher birthweight [34]. On the contrary, later solid food introduction was associated with higher child BMIz at preschool even after adjusting for covariates. Additional studies are needed to examine various maternal and child behaviors related to later solid food introduction in order to better understand and explain the association between later solid food introduction and higher BMIz at preschool among.

Although early complementary feeding has been previously associated with greater adiposity during midchildhood and adolescence in a US sample [35], different complementary feeding patterns may be unique to Middle Eastern/ Arab mothers. The association between these complementary feeding practices and long-term child outcomes need further evaluation; Longitudinal and interventions studies are warranted in order to inform effective counseling guidelines and community campaigns in the region.

To our knowledge, this study was the first in the region to evaluate certain early feeding practices including the introduction of sugary drinks in a baby bottle, and to examine associations with child birthweight and BMIz at preschool. Our study also included mothers from various backgrounds and socioeconomic levels. Additionally, anthropometric measurements were collected objectively by trained researchers. Limitations of our study include the relatively small sample size which might have affected our power to detect significant associations. Our early feeding data were obtained retrospectively and might have been subjected to recall bias. Prospective cohort studies with larger sample sizes are needed in order to confirm our findings and inform cross-cultural comparisons.

## Conclusion

In summary, although the prevalence of breastfeeding initiation was relatively high in our sample of Saudi mothers, introducing sugary drinks, including fruit juice and date syrup-milk mixture in a baby bottle appears to be a common practice. Child birthweight might influence mothers’ decisions around feeding practices. Future studies are needed to further characterize these practices and identify effects on long-term child outcomes and obesity risk. Longitudinal and interventions studies can help inform counseling guidelines and community campaigns in order to improve early feeding practices in the region.

## Data Availability

The data that support the findings of this study are available on request from the corresponding author, Dr. Rana Mosli. The data are not publicly available due to privacy or ethical restrictions.
